# A survey of ctenid spiders (Araneae, Ctenidae) from Xishuangbanna Tropical Botanical Garden, Yunnan, China

**DOI:** 10.3897/BDJ.10.e87597

**Published:** 2022-06-27

**Authors:** Chang Chu, Ying Lu, Zhiyuan Yao, Shuqiang Li

**Affiliations:** 1 College of Life Science, Shenyang Normal University, Shenyang, China College of Life Science, Shenyang Normal University Shenyang China; 2 Institute of Zoology, Chinese Academy of Sciences, Beijing, China Institute of Zoology, Chinese Academy of Sciences Beijing China

**Keywords:** biodiversity, morphology, new record, new species, taxonomy

## Abstract

**Background:**

*Amauropelma* Raven, Stumkat & Gray, 2001 currently contains 24 species. It is distributed in Australia, India, Indonesia, Laos and Malaysia. This genus has not been found in China. *Ctenus* Walckenaer, 1805 comprises 213 known species. This genus is distributed worldwide. Currently, only two species, *Ctenuslishuqiang* Jäger, 2012 and *Ctenusyaeyamensis* Yoshida, 1998 are known to occur in China.

**New information:**

Three new species of ctenid spiders are described from Xishuangbanna Tropical Botanical Garden in Yunnan Province, China: *Amauropelmayunnan* sp. nov., *Ctenusbanna* sp. nov. and *Ctenusyulin* sp. nov. *Amauropelma* and *Ctenusrobustus* Thorell, 1897 are reported from China for the first time.

## Introduction

Ctenidae Keyserling, 1877 is constituted by small to large wandering spiders (4–40 mm total body length). They are nocturnal spiders, most of them living in the lower stratum of those forests, on the ground or in the leaf litter (Höfer et al. 1994, Jocqué and Dippenaar-Schoeman 2006), whilst a few species are arboreal and cave-dwelling (Polotow and Brescovit 2008, Miller and Rahmadi 2012). The family has a worldwide distribution, but is more abundant in tropical forests in the Americas and Africa, now containing 48 genera and 527 species (Silva-Dávila 2003, Jocqué and Dippenaar-Schoeman 2006, World Spider Catalog 2022) and composed of five subfamilies: Acantheinae Simon, 1897, Acanthocteninae Simon, 1897, Calocteninae Simon, 1897, Cteninae Keyserling, 1877 and Viridasiinae Lehtinen, 1967 (Silva-Dávila 2003, Polotow and Brescovit 2014). Ctenidae is represented in China by 10 species, belonging to four genera: *Anahita* Karsch, 1879 (6 spp.), *Ctenus* Walckenaer, 1805 (2 spp.), *Leptoctenus* L. Koch, 1878 (1 sp.) and *Sinoctenus* Marusik, Zhang & Omelko, 2012 (1 sp.) (Li et al. 2014). *Ctenus* Walckenaer, 1805 under the subfamily Cteninae is the largest genus in Ctenidae, with 213 species distributed worldwide (Polotow and Brescovit 2014, World Spider Catalog 2022). However, only two species, *C.lishuqiang* Jäger, 2012 and *C.yaeyamensis* Yoshida, 1998 are known to occur in China. *Amauropelma* Raven, Stumkat & Gray, 2001 is a small ctenid genus and belongs to the subfamily Cteninae that currently contains 24 species (Polotow and Brescovit 2014, World Spider Catalog 2022). The genus is distributed in Australia (16 spp.), India (3 spp.), Indonesia (1 sp.), Laos (3 spp.) and Malaysia (1 sp.).

In China, 164,182 species of animals have been catalogued (Liu et al. 2022). Amongst them, 5,252 spider species belonging to 827 genera and 69 families were reported, accounting for only about 5% of the entire Chinese spider fauna (Li et al. 2021, Yao and Li 2021). Through the research on spider diversity in recent years, we predict that a large amount of spider diversity in China is yet to be discovered (Li et al. 2021, Yao et al. 2021, Liu et al. 2022, Lu et al. 2022). This is exemplified by previous research on spiders in Xishuangbanna, China. Xishuangbanna is located in southwest Yunnan, China. It is considered a critical area for biogeography and belongs to the Indo-Burma biodiversity hotspot. The Xishuangbanna Tropical Botanical Garden (XTBG), Chinese Academy of Sciences is considered one of the most significant tropical rainforest nature reserves in Xishuangbanna (Myers 1988, Yao et al. 2018). Before 2006, less than 50 species of spiders were reported in Xishuangbanna area, but by the end of 2020, more than 800 species of spiders have been reported in Xishuangbanna Tropical Botanical Garden of the Chinese Academy of Sciences, a 11 km^2^ peninsular area, which is the region with the most spider species on Earth (Li 2021, Yao and Li 2021). However, the family Ctenidae has not been found in Xishuangbanna. In this paper, three new species of two genera are described and illustrated. In addition, the genus *Amauropelma* and the species *Ctenusrobustus* Thorell, 1897 are reported from China for the first time.

## Materials and methods

Specimens were examined and measured with a Leica M205 C stereomicroscope. Left male pedipalps were photographed. Epigynes were photographed before dissection. Vulvae were treated in a 10% warm solution of potassium hydroxide (KOH) to dissolve soft tissues before illustration. Images were captured with a Canon EOS 750D wide zoom digital camera (24.2 megapixels) mounted on the stereomicroscope mentioned above and assembled using Helicon Focus 3.10.3 image stacking software (Khmelik et al. 2005). All measurements are given in millimetres (mm). Palp and leg measurements are shown as: total length (femur, patella, tibia, metatarsus, tarsus). Leg podomeres were measured on their dorsal side. The distribution map was generated with ArcGIS 10.2 (ESRI Incorporated Company). References to figures in the cited papers are listed in lowercase (figs.); figures from this paper are noted with a capital letter (Fig.). The specimens studied are preserved in 75% ethanol and deposited in the Institute of Zoology, Chinese Academy of Sciences (IZCAS) in Beijing, China.

Size classes are used according to Jäger (2012), total lengths: small (< 10 mm), medium (10–20 mm), large (20–30 mm), very large (> 30 mm). Palp and leg claw dentition is given according to terminology in Jäger (2012). Arising points of the embolus, median apophysis and conductor in male palps are given as clock-positions of the left palp in a ventral view. Spination pattern is given in two different formulae: in leg patellae and palp, the sum of all spines is listed for the prolateral, dorsal, retrolateral and ventral sides and, when ventral spines are absent, only three digits are listed (Davies 1994, Jäger 2012). In other leg segments, spine positions are given from proximal to distal on each side (prolateral, dorsal, retrolateral, ventral, if present) following Petrunkevitch (1925) and Jäger (2012). Leg formula is given as order of legs according to their length (femur to tarsus measured) in Arabic numbers, for example, 4123. For cheliceral teeth, large and small teeth are separated by “+”, for example, 4 + 1, meaning 4 large and 1 small teeth.

Terminology and taxonomic descriptions follow Jäger (2012). The following abbreviations are used in the descriptions: ALE = anterior lateral eye, AME = anterior median eye, AW = anterior width of prosoma, OL = opisthosoma length, OW = opisthosoma width, PL = length of dorsal shield of prosoma, PLE = posterior lateral eye, PME = posterior median eye, PW = width of dorsal shield of prosoma; used in the illustrations: C = conductor, d = dorsal, E = embolus, ET = epigynal teeth, FD = fertilisation duct, IF = internal fold, p = prolateral, PCB = prolateral cymbial bulge, r = retrolateral, RCB = retrolateral cymbial bulge, RPA = retrolateral patellar apophysis, RTA = retrolateral tibial apophysis, SP = spermathecae, SS = slit sensillum, TA = tegular apophysis, v = ventral.

## Taxon treatments

### Amauropelma yunnan

Yao & Li sp. n.

A36308BD-C752-5B3F-AE19-5C9A918346A1

DC46CB3C-CCC7-48B9-814F-C2EF7D15B709

#### Materials

- **Type status:** Holotype. **Occurrence:** recordedBy: Guo Zheng; individualCount: 1; sex: male; lifeStage: adult; **Taxon:** order: Araneae; family: Ctenidae; genus: Amauropelma; **Location:** country: China; stateProvince: Yunnan; municipality: Xishuangbanna; locality: Mengla County; verbatimLocality: Menglun Town, Xishuangbanna Tropical Botanical Garden, *Paramicheliabaillonii* plantation (about 20 yr.); verbatimElevation: 608 ± 11 m a.s.l.; verbatimLatitude: 21°54.200'N; verbatimLongitude: 101°16.923'E; **Event:** samplingProtocol: pitfall traps; year: 2007; month: 3; day: 1–15; **Record Level:** institutionCode: IZCAS-Ar 43141
- **Type status:** Paratype. **Occurrence:** recordedBy: Guo Zheng; individualCount: 1; sex: male; lifeStage: adult; **Taxon:** order: Araneae; family: Ctenidae; genus: Amauropelma; **Location:** country: China; stateProvince: Yunnan; municipality: Xishuangbanna; locality: Mengla County; verbatimLocality: Menglun Town, Xishuangbanna Tropical Botanical Garden, Rubber plantation (about 20 yr.); verbatimElevation: 586 ± 9 m a.s.l.; verbatimLatitude: 21°54.498'N; verbatimLongitude: 101°16.326'E; **Event:** samplingProtocol: pitfall traps; year: 2007; month: 2; day: 16–28; **Record Level:** institutionCode: IZCAS-Ar 43142
- **Type status:** Paratype. **Occurrence:** recordedBy: Guo Zheng; individualCount: 1; sex: male; lifeStage: adult; **Taxon:** order: Araneae; family: Ctenidae; genus: Amauropelma; **Location:** country: China; stateProvince: Yunnan; municipality: Xishuangbanna; locality: Mengla County; verbatimLocality: Menglun Town, Xishuangbanna Tropical Botanical Garden, Rubber plantation (about 20 yr.); verbatimElevation: 586 ± 9 m a.s.l.; verbatimLatitude: 21°54.498'N; verbatimLongitude: 101°16.326'E; **Event:** samplingProtocol: pitfall traps; year: 2007; month: 3; day: 1–15; **Record Level:** institutionCode: IZCAS-Ar 43143
- **Type status:** Paratype. **Occurrence:** recordedBy: Guo Zheng; individualCount: 1; sex: female; lifeStage: adult; **Taxon:** order: Araneae; family: Ctenidae; genus: Amauropelma; **Location:** country: China; stateProvince: Yunnan; municipality: Xishuangbanna; locality: Mengla County; verbatimLocality: Menglun Town, Xishuangbanna Tropical Botanical Garden, Rubber-Tea plantation (about 20 yr.); verbatimElevation: 569 ± 11 m a.s.l.; verbatimLatitude: 21°54.463'N; verbatimLongitude: 101°15.978'E; **Event:** samplingProtocol: pitfall traps; year: 2007; month: 3; day: 16–31; **Record Level:** institutionCode: IZCAS-Ar 43144
- **Type status:** Paratype. **Occurrence:** recordedBy: Guo Zheng; individualCount: 1; sex: female; lifeStage: adult; **Taxon:** order: Araneae; family: Ctenidae; genus: Amauropelma; **Location:** country: China; stateProvince: Yunnan; municipality: Xishuangbanna; locality: Mengla County; verbatimLocality: Menglun Town, Xishuangbanna Tropical Botanical Garden, *Paramicheliabaillonii* plantation (about 20 yr.); verbatimElevation: 556 ± 11 m a.s.l.; verbatimLatitude: 21°54.772'N; verbatimLongitude: 101°16.043'E; **Event:** samplingProtocol: pitfall traps; year: 2007; month: 4; day: 16–30; **Record Level:** institutionCode: IZCAS-Ar 43145
- **Type status:** Paratype. **Occurrence:** recordedBy: Guo Zheng; individualCount: 1; sex: female; lifeStage: adult; **Taxon:** order: Araneae; family: Ctenidae; genus: Amauropelma; **Location:** country: China; stateProvince: Yunnan; municipality: Xishuangbanna; locality: Mengla County; verbatimLocality: Menglun Town, Xishuangbanna Tropical Botanical Garden, Rubber-Tea plantation (about 20 yr.); verbatimElevation: 561 ± 9 m a.s.l.; verbatimLatitude: 21°55.551'N; verbatimLongitude: 101°16.923'E; **Event:** samplingProtocol: pitfall traps; year: 2007; month: 4; day: 16–30; **Record Level:** institutionCode: IZCAS-Ar 43146

#### Description

**Male** (IZCAS-Ar 43141): PL 2.4, PW 2.1, AW 0.8, OL 2.3, OW 1.5. Eye diameters and interdistances: AME 0.05, ALE 0.07, PME 0.06, PLE 0.04, AME–AME 0.04, AME–ALE 0.05, PME–PME 0.08, PME–PLE 0.12, AME–PME 0.07, ALE–PLE 0.06, clypeus AME 0.04, clypeus ALE 0.06. Palp and leg measurements: palp 2.7 (0.8, 0.4, 0.4, -, 1.1), I 6.4 (1.6, 0.9, 1.8, 1.3, 0.8), II 5.3 (1.5, 0.8, 1.2, 1.1, 0.7), III 5.0 (1.4, 0.8, 1.0, 1.1, 0.7), IV 7.2 (1.7, 0.9, 1.7, 1.9, 1.0). Leg formula 4123. Spination of palp and legs: palp 130, 000, 1010; femora I p012, d122, II p012, d111, r011, III p012, d111, r012, IV p002, d111, r112; patellae I–II 000, III–IV 001; tibiae I p010, r010, v22222, II p010, r010, v22222, III p111, d111, r111, v222, IV p111, d111, r111, v222; metatarsi I p010, r010, v222, II p010, r010, v222, III p112, d010, r112, v222, IV p112, d010, r122, v322. Chelicerae with 3 promarginal, 4 + 1 retromarginal teeth, without denticles. Retromargin of chelicerae close to fang base with 1 bristle. Tarsi and metatarsi without scopula. Claw tufts arising separately, but intermingled distally. Leg claws I with 3(2) and II with 3(1) secondary teeth. Position of tarsal organ: I 0.66, II 0.54, III 0.49, IV 0.75.

Palp (Fig. 2A–C). Patella with distinct retrolateral apophysis. RTA protruding at an almost right angle from tibia in ventral view, with two short apices, both dorso-distal. Cymbium tip slightly conical, prolatero-proximally with distinct extension. Embolus arising in a 6.30-o’clock-position from tegulum, with a distinct membranous seam. Conductor arising in a 12-o’clock-position from tegulum, bending retrolaterally. Tegular apophysis situated subcentrally on tegulum, moderately excavated at its dorsal side.

Colour (Fig. 3C and D). Yellowish-brown. Dorsal prosoma with eyes marked with black rings, faint radial markings, appearing as indistinct longitudinal bands, narrow dark margin indistinct, fovea distinct, reddish-brown. Chelicerae same colour as dorsal prosoma. Sternum, labium, gnathocoxae, ventral coxae yellowish-brown without pattern. Legs yellowish-brown. Dorsal and lateral opisthosoma yellowish with patches. Ventral opisthosoma yellowish without pattern, except for around spinnerets.

**Female** (IZCAS-Ar 43144): PL 2.4, PW 1.8, AW 1.0, OL 2.5, OW 1.6. Eye diameters and interdistances: AME 0.06, ALE 0.11, PME 0.06, PLE 0.05, AME–AME 0.06, AME–ALE 0.07, PME–PME 0.10, PME–PLE 0.17, AME–PME 0.06, ALE–PLE 0.09, clypeus AME 0.04, clypeus ALE 0.08. Palp and leg measurements: palp 2.4 (0.8, 0.4, 0.5, -, 0.7), I 6.0 (1.7, 0.9, 1.5, 1.2, 0.7), II 5.6 (1.5, 0.9, 1.3, 1.2, 0.7), III 5.0 (1.4, 0.7, 1.1, 1.1, 0.7), IV 7.8 (2.0, 0.9, 1.8, 2.0, 1.1). Leg formula 4123. Spination of palp and legs: palp 130, 100, 221, 322; femora I p010, d111, II p111, d133, III p011, d111, r011, IV p001, d212, r001; patellae I–II 000, III–IV 100; tibiae I–II v22222, III p11, d111, r11, v222, IV p111, d111, r111, v322; metatarsi I–II v222, III p112, d010, r112, v222, IV p112, d010, r112, v222. Chelicerae with 3 promarginal, 4 retromarginal teeth, without denticle. Retromargin of chelicerae close to fang base with 1 bristle. Tarsi and metatarsi without scopula. Claw tufts arising separately, but intermingled distally. Palpal claw with 4 secondary teeth, leg claws I with 3, II with 2 and IV with 1 secondary teeth. Position of tarsal organ: I 0.58, II 0.54, III 0.50, IV 0.76.

Copulatory organ (Fig. 3A and B). Epigynal field with two slit sense organs anterior to epigynal plate. Epigynal plate width/length: 9.6/4.9, 9.8/4.7; anterior width/posterior width: 9.6/3.1, 9.8/3.4; posterior part with distinct concave lateral margins. Distal part of lateral teeth posteriorad. Internal duct system with round spermathecae fully visible, separated from each other by more than their diameter; fertilisation ducts laminar, mediad.

Colour (Fig. 3E and F). As in male, except for: chelicerae reddish-brown. Dorsal, lateral and ventral opisthosoma yellowish without pattern.

**Variation**: Males (IZCAS-Ar 43142, Ar 43143): PL 2.4–2.7, OL 2.3–2.7. Females (IZCAS-Ar 43145, Ar 43146): PL 1.9–2.2, OL 2.3–2.4.

#### Diagnosis

Small Ctenidae (total length male 4.7–5.4, female 4.2–4.9). The species resembles *A.staschi* Jäger, 2012 (see Jäger 2012: figs. 187–192) by having similar male tegular apophysis, tegulum and RTA (Fig. 2A–C), but can be easily distinguished by the embolus arising in a 6.30-o’clock-position from tegulum (Fig. 2B; 9-o’clock-position in *A.staschi*), by the conductor arising in a 12-o’clock-position from tegulum (Fig. 2B; 1 to 1.30-o’clock-position in *A.staschi*), by the tegulum with sclerotised, triangular distal apophysis (Fig. 2B; absent in *A.staschi*) and by the patella of palp with distinct retrolateral apophysis (Fig. 2B; without distinct apophysis, retrolateral side slightly swollen in *A.staschi*).

#### Etymology

The specific name refers to the type locality and is a noun in apposition.

#### Distribution

**Figure 1.**
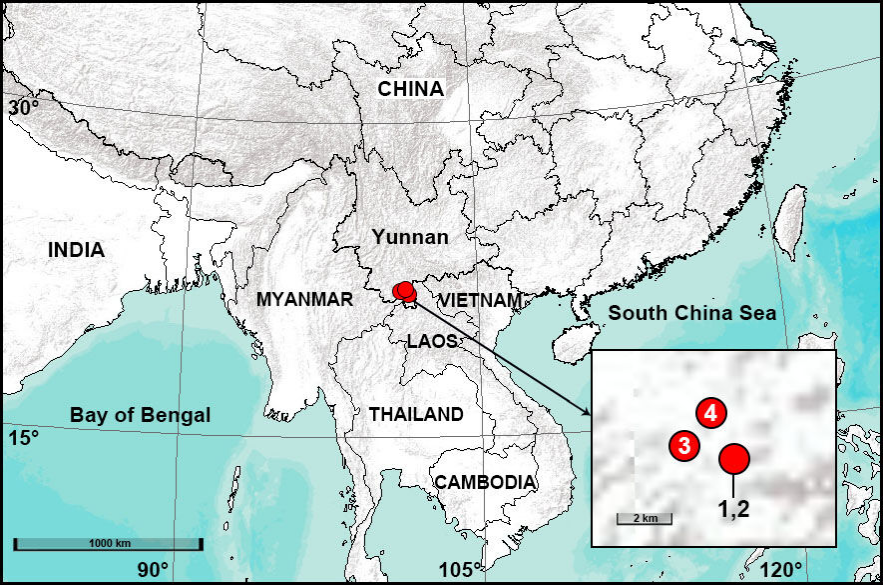
Distribution records of new and newly-recorded ctenid species from Xishuangbanna Tropical Botanical Garden in Yunnan Province, China. 1. *Amauropelmayunnan* sp. nov.; 2. *Ctenusbanna* sp. nov.; 3. *C.robustus* Thorell, 1897; 4. *C.yulin* sp. nov.

**Figure 2.**
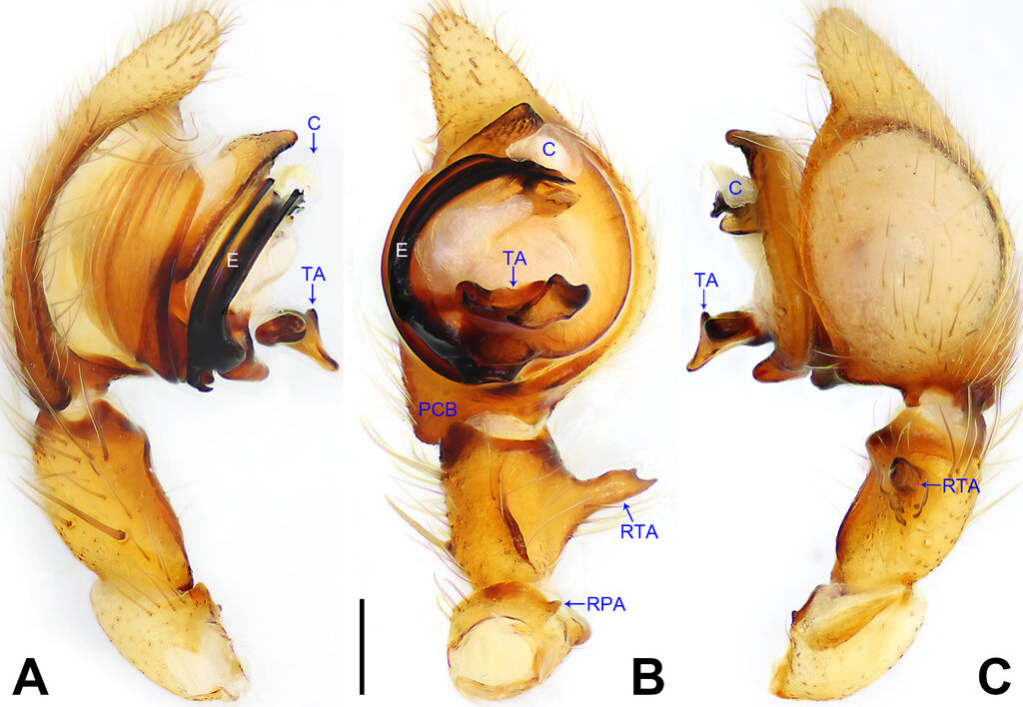
*Amauropelmayunnan* sp. nov., holotype male. **A** Palp, prolateral view; **B** Palp, ventral view; **C** Palp, retrolateral view. C = conductor, E = embolus, PCB = prolateral cymbial bulge, RPA = retrolateral patellar apophysis, RTA = retrolateral tibial apophysis, TA = tegular apophysis. Scale bar: 0.20 mm (A–C).

**Figure 3.**
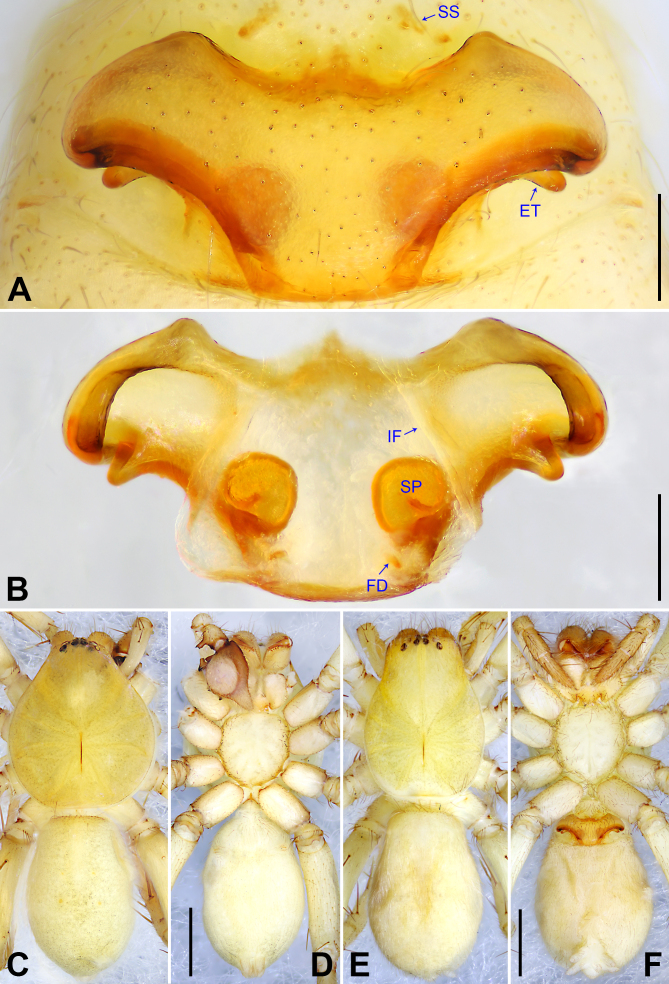
*Amauropelmayunnan* sp. nov. **A** Paratype female, epigyne, ventral view; **B** Paratype female, vulva, dorsal view; **C** Holotype male, habitus, dorsal view; **D** Holotype male, habitus, ventral view; **E** Paratype female, habitus, dorsal view; **F** Paratype female, habitus, ventral view. ET = epigynal teeth, FD = fertilisation duct, IF = internal fold, SP = spermathecae, SS = slit sensillum. Scale bars: 0.20 mm (A, B), 1.00 mm (C–F).

China (Yunnan, type locality; Fig. 1).

### Ctenus banna

Yao & Li sp. n.

EE4AE4A2-443E-5823-AEEB-C316DAE1DECA

3617C057-2BD1-4ADC-8FF1-8F63B9C3A163

#### Materials

- **Type status:** Holotype. **Occurrence:** recordedBy: Guo Zheng; individualCount: 1; sex: male; lifeStage: adult; **Taxon:** order: Araneae; family: Ctenidae; genus: Ctenus; **Location:** country: China; stateProvince: Yunnan; municipality: Xishuangbanna; locality: Mengla County; verbatimLocality: Menglun Town, Xishuangbanna Tropical Botanical Garden, *Paramicheliabaillonii* plantation (about 20 yr.); verbatimElevation: 608 ± 11 m a.s.l.; verbatimLatitude: 21°54.200'N; verbatimLongitude: 101°16.923'E; **Event:** samplingProtocol: pitfall traps; year: 2007; month: 6; day: 1–15; **Record Level:** institutionCode: IZCAS-Ar 43147
- **Type status:** Paratype. **Occurrence:** recordedBy: Guo Zheng; individualCount: 1; sex: male; lifeStage: adult; **Taxon:** order: Araneae; family: Ctenidae; genus: Ctenus; **Location:** country: China; stateProvince: Yunnan; municipality: Xishuangbanna; locality: Mengla County; verbatimLocality: Menglun Town, Xishuangbanna Tropical Botanical Garden, *Paramicheliabaillonii* plantation (about 20 yr.); verbatimElevation: 608 ± 11 m a.s.l.; verbatimLatitude: 21°54.200'N; verbatimLongitude: 101°16.923'E; **Event:** samplingProtocol: pitfall traps; year: 2007; month: 6; day: 1–15; **Record Level:** institutionCode: IZCAS-Ar 43148
- **Type status:** Paratype. **Occurrence:** recordedBy: Guo Zheng; individualCount: 1; sex: female; lifeStage: adult; **Taxon:** order: Araneae; family: Ctenidae; genus: Ctenus; **Location:** country: China; stateProvince: Yunnan; municipality: Xishuangbanna; locality: Mengla County; verbatimLocality: Menglun Town, Xishuangbanna Tropical Botanical Garden, Rubber plantation (about 20 yr.); verbatimElevation: 585 ± 10 m a.s.l.; verbatimLatitude: 21°54.684'N; verbatimLongitude: 101°16.319'E; **Event:** samplingProtocol: pitfall traps; year: 2007; month: 7; day: 1–15; **Record Level:** institutionCode: IZCAS-Ar 43149
- **Type status:** Paratype. **Occurrence:** recordedBy: Guo Zheng; individualCount: 1; sex: female; lifeStage: adult; **Taxon:** order: Araneae; family: Ctenidae; genus: Ctenus; **Location:** country: China; stateProvince: Yunnan; municipality: Xishuangbanna; locality: Mengla County; verbatimLocality: Menglun Town, Xishuangbanna Tropical Botanical Garden, *Paramicheliabaillonii* plantation (about 20 yr.); verbatimElevation: 608 ± 11 m a.s.l.; verbatimLatitude: 21°54.200'N; verbatimLongitude: 101°16.923'E; **Event:** samplingProtocol: pitfall traps; year: 2007; month: 6; day: 1–15; **Record Level:** institutionCode: IZCAS-Ar 43150

#### Description

**Male** (IZCAS-Ar 43147): PL 6.7, PW 5.3, AW 2.5, OL 5.8, OW 4.0. Eye diameters and interdistances: AME 0.25, ALE 0.22, PME 0.29, PLE 0.26, AME–AME 0.14, AME–ALE 0.33, PME–PME 0.20, PME–PLE 0.39, AME–PME 0.16, ALE–PLE 0.15, clypeus AME 0.17, clypeus ALE 0.62. Palp and leg measurements: palp 7.1 (2.6, 1.0, 1.3, -, 2.2), I 21.3 (5.6, 2.6, 5.8, 5.4, 1.9), II 19.3 (5.4, 2.4, 4.8, 4.9, 1.8), III 15.8 (4.4, 2.2, 3.2, 4.4, 1.6), IV 23.4 (5.8, 2.5, 5.8, 7.4, 1.9). Leg formula 4123. Spination of palp and legs: palp 141, 100, 1010; femora I p121, d111, r112, II p122, d121, r212, III p112, d111, r012, IV p112, d111, r012; patellae 101; tibiae I p011, d111, r110, v22222, II p100, d101, r102, v22222, III–IV p11, d111, r11, v222; metatarsi I–II p111, r111, v223, III p112, r112, v222, IV p112, d010, r112, v2222. Chelicerae with 3 promarginal, 4 retromarginal teeth and with elongated patch of 23 tiny denticles along entire cheliceral furrow. Retromargin of chelicerae close to fang base with 4–5 bristles. Sparse scopula restricted almost entirely to tarsi, only metatarsi I–II with sparse scopula hairs. Right leg claw I with 4 secondary teeth. Position of tarsal organ: I 1.82, II1.46, III 1.19, IV 1.56.

**Figure 4.**
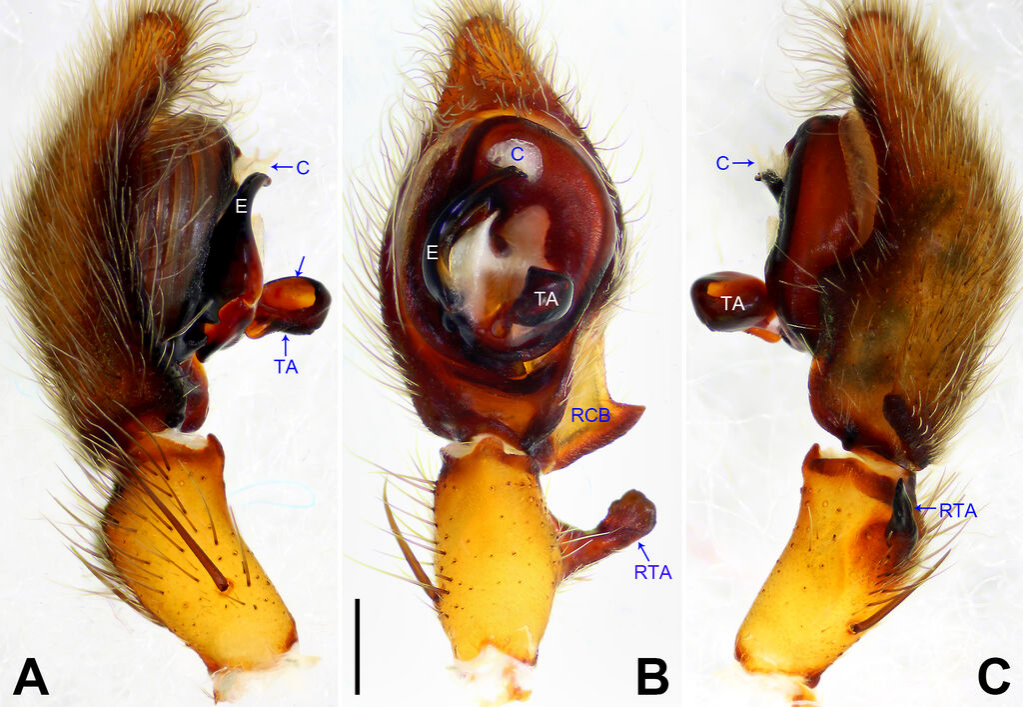
*Ctenusbanna* sp. nov., holotype male. **A** Palp, prolateral view, arrow points at excavation; **B** Palp, ventral view; **C** Palp, retrolateral view. C = conductor, E = embolus, RCB = retrolateral cymbial bulge, RTA = retrolateral tibial apophysis, TA = tegular apophysis. Scale bar: 0.20 mm (A–C).

Palp (Fig. 4A–C). Palpal tibia with strong RTA, with broad base and blunt tip. Cymbium tip slightly conical, retro-proximally with distinct pointed extension. Embolus arising at 7.30-o’clock-position, short and with three apophyses, its tip situated in distal half of tegulum. Conductor arising at 12-o’clock-position distally, partly fused with tegulum. Tegular apophysis arising at 6-o’clock-position from tegulum, distinctly excavated on prolateral side (arrowed in Fig. 4A).

**Figure 5.**
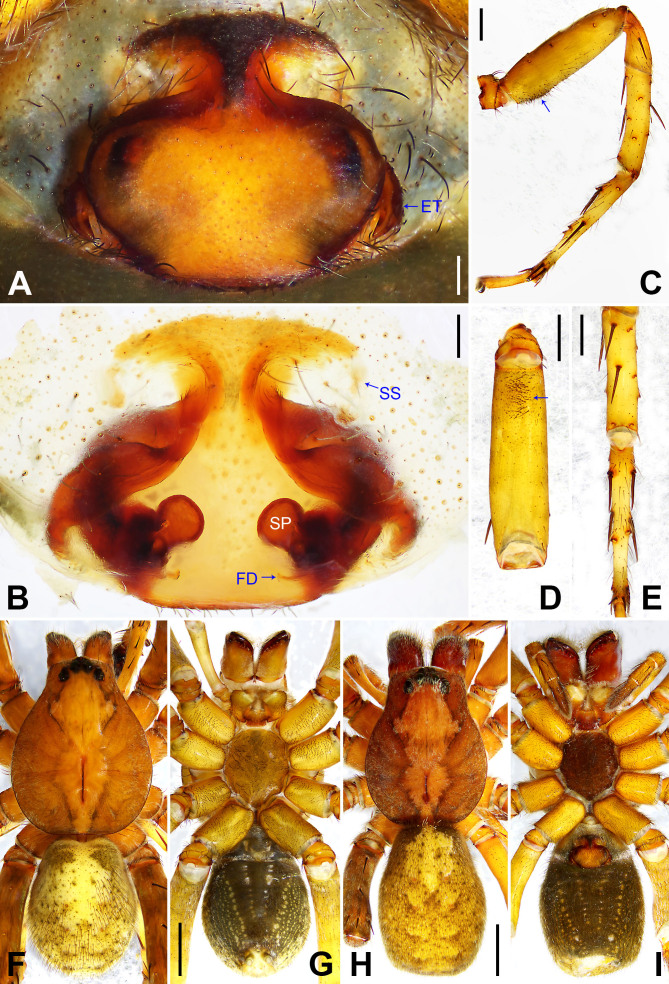
*Ctenusbanna* sp. nov. **A** Paratype female, epigyne, ventral view; **B** Paratype female, vulva, dorsal view; **C** Holotype male, leg III, prolateral view, arrow points at hump; **D** Holotype male, femur III, ventral view, arrow points at hump; **E** Holotype male, tibia III + metatarsus III, ventral view; **F** Holotype male, habitus, dorsal view; **G** Holotype male, habitus, ventral view; **H** Paratype female, habitus, dorsal view; **I** Paratype female, habitus, ventral view. ET = epigynal teeth, FD = fertilisation duct, SP = spermathecae, SS = slit sensillum. Scale bars: 0.20 mm (A, B), 1.00 mm (C–E), 2.00 mm (F–I).

Colour (Fig. 5F and G). Reddish-brown to yellowish with darker patterns. Dorsal prosoma with light median band with characteristic widening behind eyes and some white hairs; with distinctly marked fovea and indistinct radial markings. Sternum, ventral coxae III + IV and gnathocoxae yellowish, with dark patterns, labium brown with dark patterns, coxae I + II yellowish without patterns. Chelicerae with longitudinal lines in proximal half and with dark distal half. Leg femora and patellae reddish-brown-yellowish, tibia to tarsi yellowish. Dorsal opisthosoma yellowish, mottled with dark spots. Lateral opisthosoma yellowish with dark spots. Ventral opisthosoma black with white patches; epiandrium and muscle sigilla light. Anterior lateral spinnerets dark, posterior lateral and median spinnerets and anal tubercle light.

**Female** (IZCAS-Ar 43149): PL 6.0, PW 4.7, AW 3.0, OL 5.8, OW 4.1. Eye diameters and interdistances: AME 0.23, ALE 0.16, PME 0.31, PLE 0.27, AME–AME 0.21, AME–ALE 0.40, PME–PME 0.25, PME–PLE 0.46, AME–PME 0.16, ALE–PLE 0.20, clypeus AME 0.13, clypeus ALE 0.52. Palp and leg measurements: palp 5.9 (2.0, 1.1, 1.2, -, 1.6), I 13.5 (3.6, 2.3, 3.5, 2.9, 1.2), II 13.3 (3.6, 2.3, 3.3, 3.0, 1.1), III 11.3 (3.4, 1.7, 2.3, 2.8, 1.1), IV 16.0 (3.9, 2.2, 3.8, 4.6, 1.5). Leg formula 4123. Spination of palp and legs: palp 131, 100, 1111, 2101; femora I p021, d111, r111, II p112, d111, r 111, III p212, d111, r112, IV p101, d111, r001; patellae I–II 000, III–IV p010, r010; tibiae I–II v22222, III p11, d110, r11, v222, IV p11, d111, r11, v222; metatarsi I–II v222, III p112, r112, v222, IV p112, d010, r112, v2222. Chelicerae with 3 promarginal, 4 retromarginal teeth and with elongated patch of 22 tiny denticles along entire cheliceral furrow. Retromargin of chelicerae close to fang base with 5 thin bristles. Sparse scopula restricted almost entirely to tarsi, only metatarsi I–II with sparse scopula hairs. Palpal claw with 5 secondary teeth. Right leg claw I with 2 secondary teeth. Position of tarsal organ: I 1.01, II 0.94, III 0.89, IV 1.16.

Copulatory organ (Fig. 5A and B). Epigynal field with two separate anterio-lateral patches, close to these patches two slit sense organs. Median plate narrowed anteriorly, widening medially and narrowing posteriorly again, posteriorly with subparallel margins, lateral teeth situated at widest part. Posterior epigyne with indistinct lateral furrows. Internal duct system with two large lateral folds running diagonally from medially to laterally. Spermathecae separated from each other by more than their diameter, with a small round chamber between spermathecae and fertilisation ducts, the latter pointing medially.

Colour (Fig. 5H and I). As in male, except for: sternum dark brown, ventral coxa IV yellowish with dark pattens, labium and gnathocoxae brown. Chelicerae reddish-brown.

**Variation**: Paratype male (IZCAS-Ar 43148): PL 5.7, OL 4.7. Second female paratype (IZCAS-Ar 43150): PL 6.4, OL 6.8.

#### Diagnosis

Medium Ctenidae (total length male 10.4–12.5, female 11.8–13.2). The species resembles *C.theodorianum* Jäger, 2012 (see Jäger 2012: figs. 92–115) by having similar male RTA (Fig. 4B) and female copulatory organ (Fig. 5A and B), but can be easily distinguished by tegular apophysis undivided in ventral view (Fig. 4B; divided in two parts in *C.theodorianum*) and with distinct excavation prolaterally (arrowed in Fig. 4A; indistinct excavation on dorsal side in *C.theodorianum*), by the embolus with three apophyses, its tip situated in distal half of tegulum (Fig. 4B; embolus with two apophyses, its tip situated almost centrally in *C.theodorianum*), by the leg III in males with indistinct and not strongly swollen ventral hump covered by small stout spines on the proximal femur (arrowed in Fig. 5C and D; more distinctly swollen in *C.theodorianum*) and by the tibia III in males without ventral cone-shaped humps (Fig. 5C and E; present in *C.theodorianum*).

#### Etymology

The specific name refers to the type locality (Banna is a short name for Xishuangbanna) and is a noun in apposition.

#### Distribution

China (Yunnan, type locality, Fig. 1).

### Ctenus robustus

Thorell, 1897

6E44AB41-C757-59DC-B632-86AE2BD4033E

#### Materials

- **Type status:** Other material. **Occurrence:** recordedBy: Guo Zheng; individualCount: 1; sex: male; lifeStage: adult; **Taxon:** order: Araneae; family: Ctenidae; genus: Ctenus; **Location:** country: China; stateProvince: Yunnan; municipality: Xishuangbanna; locality: Mengla County; verbatimLocality: Menglun Town, Xishuangbanna Tropical Botanical Garden, Rubber plantation (about 20 yr.); verbatimElevation: 569 ± 11 m a.s.l.; verbatimLatitude: 21°54.463'N; verbatimLongitude: 101°15.978'E; **Event:** samplingProtocol: searching; year: 2007; month: 7; day: 10–20; **Record Level:** institutionCode: IZCAS-Ar 43151
- **Type status:** Other material. **Occurrence:** recordedBy: Guo Zheng; individualCount: 1; sex: male; lifeStage: adult; **Taxon:** order: Araneae; family: Ctenidae; genus: Ctenus; **Location:** country: China; stateProvince: Yunnan; municipality: Xishuangbanna; locality: Mengla County; verbatimLocality: Menglun Town, Xishuangbanna Tropical Botanical Garden, Rubber plantation (about 20 yr.); verbatimElevation: 569 ± 11 m a.s.l.; verbatimLatitude: 21°54.463'N; verbatimLongitude: 101°15.978'E; **Event:** samplingProtocol: pitfall traps; year: 2007; month: 7; day: 1–15; **Record Level:** institutionCode: IZCAS-Ar 43152
- **Type status:** Other material. **Occurrence:** recordedBy: Guo Zheng; individualCount: 1; sex: male; lifeStage: adult; **Taxon:** order: Araneae; family: Ctenidae; genus: Ctenus; **Location:** country: China; stateProvince: Yunnan; municipality: Xishuangbanna; locality: Mengla County; verbatimLocality: Menglun Town, Xishuangbanna Tropical Botanical Garden, Secondary tropical seasonal rain forest; verbatimElevation: 598 ± 17 m a.s.l.; verbatimLatitude: 21°55.428'N; verbatimLongitude: 101°16.441'E; **Event:** samplingProtocol: searching; year: 2007; month: 5; day: 19–26; **Record Level:** institutionCode: IZCAS-Ar 43153

#### Description

see Jäger (2012).

#### Diagnosis

see Jäger (2012).

#### Distribution

Myanmar (type locality), Laos, China (Yunnan, Fig. 1).

### Ctenus yulin

Yao & Li sp. n.

A4CF148B-ED05-5776-9E10-680ECEAEE3A1

87D1275C-26AA-4D00-8077-69DA993C2DE3

#### Materials

- **Type status:** Holotype. **Occurrence:** recordedBy: Guo Zheng; individualCount: 1; sex: male; lifeStage: adult; **Taxon:** order: Araneae; family: Ctenidae; genus: Ctenus; **Location:** country: China; stateProvince: Yunnan; municipality: Xishuangbanna; locality: Mengla County; verbatimLocality: Menglun Town, Xishuangbanna Tropical Botanical Garden, Primary tropical seasonal rain forest; verbatimElevation: 558 ± 17 m a.s.l.; verbatimLatitude: 21°55.035'N; verbatimLongitude: 101°16.500'E; **Event:** samplingProtocol: searching; year: 2007; month: 4; day: 4–11; **Record Level:** institutionCode: IZCAS-Ar 43154
- **Type status:** Paratype. **Occurrence:** recordedBy: Guo Zheng; individualCount: 1; sex: male; lifeStage: adult; **Taxon:** order: Araneae; family: Ctenidae; genus: Ctenus; **Location:** country: China; stateProvince: Yunnan; municipality: Xishuangbanna; locality: Mengla County; verbatimLocality: Menglun Town, Xishuangbanna Tropical Botanical Garden, Primary tropical seasonal rain forest; verbatimElevation: 558 ± 17 m a.s.l.; verbatimLatitude: 21°55.035'N; verbatimLongitude: 101°16.500'E; **Event:** samplingProtocol: searching; year: 2007; month: 4; day: 4–11; **Record Level:** institutionCode: IZCAS-Ar 43155
- **Type status:** Paratype. **Occurrence:** recordedBy: Guo Zheng; individualCount: 1; sex: female; lifeStage: adult; **Taxon:** order: Araneae; family: Ctenidae; genus: Ctenus; **Location:** country: China; stateProvince: Yunnan; municipality: Xishuangbanna; locality: Mengla County; verbatimLocality: Menglun Town, Xishuangbanna Tropical Botanical Garden, Primary tropical seasonal rain forest; verbatimElevation: 558 ± 17 m a.s.l.; verbatimLatitude: 21°55.035'N; verbatimLongitude: 101°16.500'E; **Event:** samplingProtocol: searching; year: 2007; month: 3; day: 19–26; **Record Level:** institutionCode: IZCAS-Ar 43156
- **Type status:** Paratype. **Occurrence:** recordedBy: Guo Zheng; individualCount: 1; sex: female; lifeStage: adult; **Taxon:** order: Araneae; family: Ctenidae; genus: Ctenus; **Location:** country: China; stateProvince: Yunnan; municipality: Xishuangbanna; locality: Mengla County; verbatimLocality: Menglun Town, Xishuangbanna Tropical Botanical Garden, Primary tropical seasonal rain forest; verbatimElevation: 558 ± 17 m a.s.l.; verbatimLatitude: 21°55.035'N; verbatimLongitude: 101°16.500'E; **Event:** samplingProtocol: searching; year: 2007; month: 3; day: 19–26; **Record Level:** institutionCode: IZCAS-Ar 43157

#### Description

**Male** (IZCAS-Ar 43154): PL 3.6, PW 2.8, AW 1.4, OL 3.4, OW 2.2. Eye diameters and interdistances: AME 0.15, ALE 0.13, PME 0.19, PLE 0.18, AME–AME 0.11, AME–ALE 0.24, PME–PME 0.17, PME–PLE 0.26, AME–PME 0.10, ALE–PLE 0.15, clypeus AME 0.10, clypeus ALE 0.31. Palp and leg measurements: palp 4.2 (1.6, 0.6, 0.8, -, 1.2), I 12.2 (3.2, 1.4, 3.3, 3.0, 1.3), II 10.9 (3.0, 1.4, 2.8, 2.6, 1.1), III 9.7 (2.8, 1.2, 2.1, 2.5, 1.1), IV - (3.8, 1.3, 3.4, 3.9, -). Leg formula 4123. Spination of palp and legs: palp 130, 000, 101; femora I p002, d111, r011, II p012, d110, r012, III p112, d111, r112, IV p012, d111, r002; patellae I–II 000, III–IV 101; tibiae I r100, v22222, II p010, r010, v22222, III p11, d111, r11, v222, IV p111, d110, r11, v222; metatarsi I p000, r011, v223, II p011, r111, v222, III p112, d010, r112, v222, IV p112, r112, v222. Chelicerae with 3 promarginal, 4 retromarginal teeth and with elongated patch of 8 tiny denticles along entire cheliceral furrow. Retromargin of chelicerae close to fang base with 1–2 bristles. Sparse scopula restricted almost entirely to tarsi. Leg claws I with 5, II–III with 4 secondary teeth. Position of tarsal organ: I 1.02, III 0.70.

**Figure 6.**
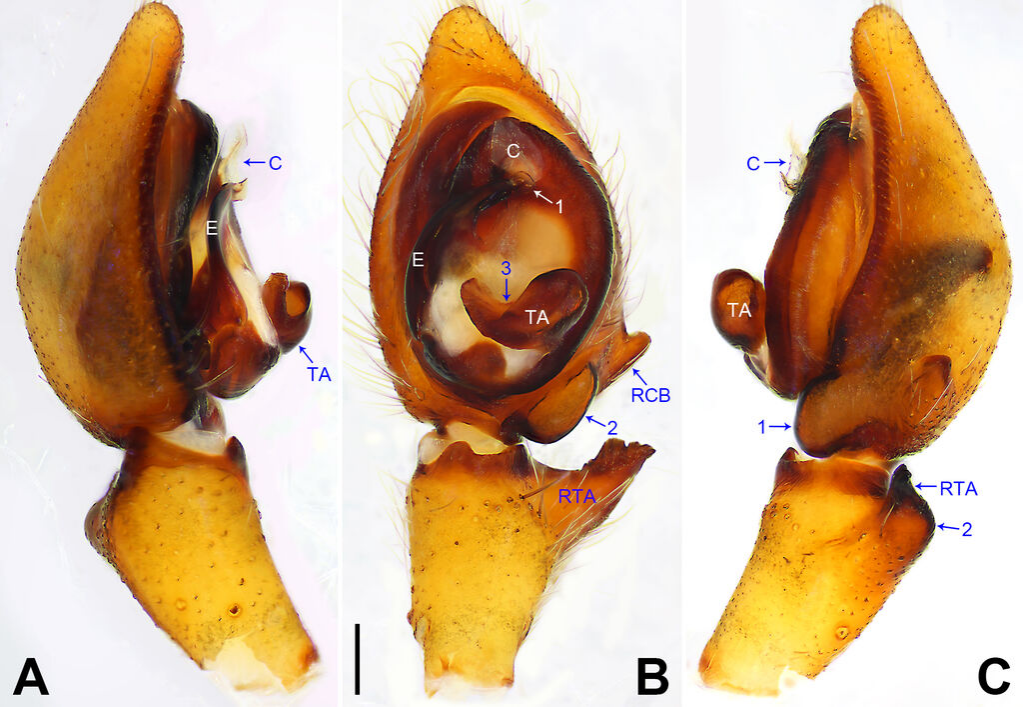
*Ctenusyulin* sp. nov., holotype male. **A** Palp, prolateral view; **B** Palp, ventral view, arrow 1 points at membranous process, arrow 2 points at protuberance, arrow 3 points at concave; **C** Palp, retrolateral view, arrow 1 points at protuberance, arrow 2 points at strongly swollen. C = conductor, E = embolus, RCB = retrolateral cymbial bulge, RTA = retrolateral tibial apophysis, TA = tegular apophysis. Scale bar: 0.20 mm (A–C).

Palp (Fig. 6A–C). Palpal tibia with strong RTA, distally with teeth and strongly swollen on proximal part (arrowed 2 in Fig. 6C). Cymbium tip slightly conical, retro-proximally with distinct pointed extension and proximally with distinct large retrolateral protuberance (arrowed 2 in Fig. 6B, arrowed 1 in Fig. 6C). Embolus arising in an 8-o’clock-position from tegulum, short, its tip situated in distal half of tegulum and with small membranous process distally (arrowed 1 in Fig. 6B). Conductor arising in a 12-o’clock-position from tegulum, almost completely transparent. Tegular apophysis arising at 6-o’clock-position from tegulum, distinctly excavated on dorsal side and strongly concave on ventral side (arrowed 3 in Fig. 6B).

**Figure 7.**
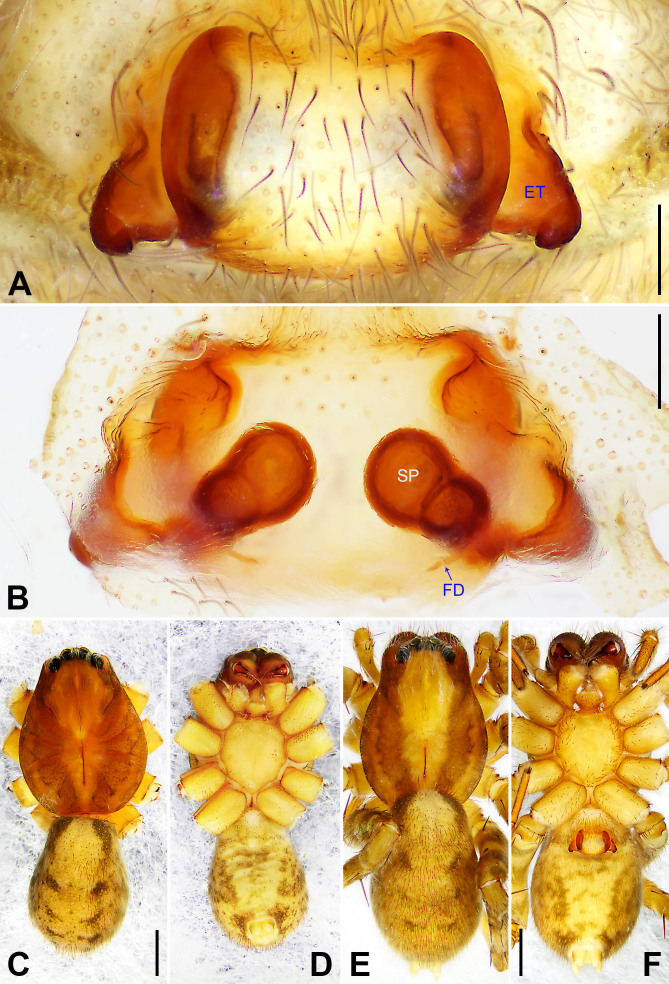
*Ctenusyulin* sp. nov. **A** Paratype female, epigyne, ventral view; **B** Paratype female, vulva, dorsal view; **C** Holotype male, habitus, dorsal view; **D** Holotype male, habitus, ventral view; **E** Paratype female, habitus, dorsal view; **F** Paratype female, habitus, ventral view. ET = epigynal teeth, FD = fertilisation duct, SP = spermathecae. Scale bars: 0.20 mm (A, B), 1.00 mm (C–F).

Colour (Fig. 7C and D). Yellowish to reddish-brown with darker patterns. Dorsal prosoma with characteristic slightly lighter median band, widened behind eyes and with distinctly marked fovea and distinct radial markings. Sternum, labium, gnathocoxae, ventral coxae yellowish, without patterns. Chelicerae reddish-brown, with longitudinal lines in proximal half and with darker distal half. Palps and legs yellowish to reddish-brown. Dorsal opisthosoma yellowish with black patches, most fused into two parallel rows. Lateral opisthosoma spotted. Ventral opisthosoma yellowish, lateral and median with darker patterns. Spinnerets light and anal tubercle light.

**Female** (IZCAS-Ar 43156): PL 3.7, PW 2.7, AW 1.9, OL 3.8, OW 2.5. Eye diameters and interdistances: AME 0.15, ALE 0.16, PME 0.20, PLE 0.23, AME–AME 0.15, AME–ALE 0.26, PME–PME 0.21, PME–PLE 0.32, AME–PME 0.13, ALE–PLE 0.16, clypeus AME 0.11, clypeus ALE 0.32. Palp and leg measurements: palp 3.5 (1.1, 0.7, 0.8, -, 0.9), I 9.2 (2.5, 1.4, 2.5, 1.9, 0.9), II 8.6 (2.3, 1.3, 2.2, 1.9, 0.9), III 8.2 (2.1, 1.2, 1.9, 2.0, 1.0), IV 11.4 (3.0, 1.3, 2.6, 3.3, 1.2). Leg formula 4123. Spination of palp and legs: palp 130, 100, 1101, 2102; femora I p002, d111, r010, II p011, d111, r010, III p111, d111, r112, IV p201, d111, r001; patellae I–II 000, III–IV 101; tibiae I–II v22222, III p11, d11, r11, v222, IV p11, d111, r11, v222; metatarsi I–II v222, III–IV p112, d010, r112, v222. Chelicerae with 3 promarginal, 4 retromarginal teeth and with elongated patch of 6 tiny denticles along entire cheliceral furrow. Retromargin of chelicerae close to fang base with 2 bristles. Sparse scopula restricted almost entirely to tarsi. Palpal claw with 7 secondary teeth, leg claws I with 5, II–III with 4 and IV with 5 secondary teeth. Position of tarsal organ: I 0.68, II 0.65, III 0.50, IV 0.65.

Copulatory organ (Fig. 7A and B). Epigynal field laterally with two separate long nearly elliptic patches. The anterior of median plate is as wide as posterior, lateral teeth nearly triangular, situated posteriorly at widest part. Internal duct system with two small lateral folds. Spermathecae separated by less than their diameter, with a large round chamber between spermathecae and fertilisation ducts, the latter pointing medially.

Colour (Fig. 7E and F). As in male, except for: dorsal prosoma with light median band and without distinct radial markings. Chelicerae dark reddish-brown, with longitudinal lines. Leg I femur with indistinct dark rings, legs II–IV femora, patellae and tibia with distinct dark rings.

**Variation**: Paratype male (IZCAS-Ar 43155): PL 3.1, OL 2.8. Second paratype female (IZCAS-Ar 43157): PL 3.9, OL 4.5.

#### Diagnosis

Small Ctenidae (total length male 5.9–7.0, female 7.5–8.4). The new species can be easily distinguished from all known congeners by the embolus with small membranous process distally (arrowed 1 in Fig. 6B), by the tegular apophysis strongly concave on ventral side (arrowed 3 in Fig. 6B), by the cymbium with distinct large proximal protuberance (arrowed 2 in Fig. 6B, arrowed 1 in Fig. 6C), by the RTA strongly swollen on proximal part (arrowed 2 in Fig. 6C) and by the vulva spermathecae large, two chambers of spermathecae nearly rounded and distinct (Fig. 7B).

#### Etymology

The specific name is a Chinese pinyin word for rainforest (yǔ lín) and is a noun in apposition.

#### Distribution

China (Yunnan, type locality, Fig. 1).

## Supplementary Material

XML Treatment for Amauropelma yunnan

XML Treatment for Ctenus banna

XML Treatment for Ctenus robustus

XML Treatment for Ctenus yulin
